# Association between the non-high-density lipoprotein cholesterol to high-density lipoprotein cholesterol ratio and peripheral artery disease in vascular surgery inpatients aged 50 and above: a retrospective cross-sectional study

**DOI:** 10.3389/fmed.2026.1739515

**Published:** 2026-01-21

**Authors:** Zhi-wei Li, Bei-hao Shi, Jie Ren, Dian Chen, Ya-qin Gong, Ke Lu, Jian Zhu

**Affiliations:** 1 Department of Vascular Surgery, Kunshan First People’s Hospital, Gusu School of Nanjing Medical University, Kunshan, Jiangsu, China; 2 Department of Vascular Surgery, Affiliated Kunshan Hospital of Jiangsu University, Suzhou, China

**Keywords:** atherosclerosis, lipid metabolism, non-high-density lipoprotein cholesterol to high-density lipoprotein cholesterol ratio, peripheral artery disease, threshold effect

## Abstract

**Background:**

Peripheral artery disease (PAD) is a major manifestation of systemic atherosclerosis and affects vascular health in older adults. Dyslipidaemia contributes significantly to PAD, but the predictive value of composite lipid indices remains unclear. The non-high-density lipoprotein cholesterol (non-HDL-C) to high-density lipoprotein cholesterol (HDL-C) ratio (NHHR) reflects the balance between atherogenic and protective lipoproteins. This study aimed to explore the association between the NHHR and PAD among vascular surgery inpatients aged ≥50 years in Kunshan, China.

**Methods:**

This retrospective cross-sectional study included 3,532 patients (aged ≥ 50 years) hospitalized at the Affiliated Kunshan Hospital of Jiangsu University, Suzhou, from December 2017 to August 2024. NHHR, calculated as (total cholesterol − HDL-C)/HDL-C, was the exposure variable; PAD, defined as PAD-like symptoms with an ankle brachial index < 0.9, was the outcome. Covariates included age, sex, lipoprotein(a) level [Lp(a)], apolipoprotein A1 level (Apo A1), alanine aminotransferase (ALT) level, neutrophil count (NEUT), hypertension status, diabetes status, smoking status, and alcohol consumption status. Multivariate logistic regression, smooth curve fitting, and threshold analyses were performed.

**Results:**

After adjustment for confounders, the NHHR was nonlinearly associated with PAD (OR = 0.77; 95% CI: 0.65–0.93; *p* = 0.006). Patients in the third NHHR quartile had the lowest PAD risk (OR = 0.51; 95% CI: 0.35–0.75; *p* < 0.001). Smooth curve fitting indicated a J-shaped relationship with a turning point around NHHR = 2.75. Below this threshold, a higher NHHR correlated with a lower PAD risk, whereas above it, the risk increased. A significant sex interaction was observed (*P* for interaction < 0.05).

**Conclusion:**

The NHHR was associated with the presence of PAD, with the evidence suggesting a nonlinear relationship and potential sex-specific differences. Given the retrospective cross-sectional design, this association does not support causal inference or strong predictive claims. The NHHR may help identify individuals who could benefit from further clinical evaluation for PAD, but prospective studies are needed to confirm its clinical relevance before its routine application.

## Introduction

Peripheral arterial disease (PAD) is a prevalent disorder of the peripheral vasculature. Its hallmark pathology is atherosclerosis in the arteries of the lower extremities, resulting in arterial stenosis or occlusion and consequent haemodynamic compromise (1, 2). Clinically, PAD often presents as intermittent claudication, specifically, ischaemic muscular pain triggered by walking and relieved by rest. Sustained ischaemia may lead to tissue loss or even gangrene in the affected limb (3, 4). The global incidence of PAD is increasing, especially in older adults, with a prevalence exceeding 18% in individuals over 60 years of age (5). Despite the availability of numerous treatment strategies, the rate of PAD recurrence remains high (6, 7), posing a major challenge and placing a substantial economic burden on health care systems. Accordingly, the identification of modifiable risk factors for PAD is crucial for its effective prevention and treatment.

The development of PAD involves a complex interplay of genetic, environmental, dietary, lifestyle, and metabolic factors. Epidemiological studies have shown links between PAD risk and multiple factors, including sex, age, race, smoking status, alcohol consumption, and comorbid conditions such as hypertension and diabetes (8–12). Among these, dyslipidaemia plays a pivotal role in PAD progression by promoting atherosclerosis in the arteries of the lower extremities (13, 14). In particular, elevated levels of low-density lipoprotein cholesterol (LDL-C) and total cholesterol have been shown to contribute significantly to PAD development (14, 15), whereas higher high-density lipoprotein cholesterol (HDL-C) levels are associated with a lower PAD risk (16, 17). These divergent effects of LDL-C and HDL-C underscore the importance of cholesterol management in the maintenance of vascular health.

Compared with individual lipid measures, the non-HDL-C to HDL-C ratio (NHHR) has recently emerged as an innovative composite lipid index that may offer a better reflection of overall atherogenic risk. Unlike isolated measures such as LDL-C or total cholesterol (TC), the NHHR simultaneously captures both atherogenic (non-HDL-C) and protective (HDL-C) lipid components, thus integrating the balance between cholesterol deposition and clearance (18). Increasing evidence indicates that compared with traditional lipid parameters, the NHHR has superior ability to predict cardiovascular and cerebrovascular risk (19, 20). In addition, the NHHR offers several practical advantages over other emerging lipid indices. First, it is simple to calculate, requiring only TC and HDL-C values that are routinely available in clinical testing. Second, the NHHR is stable across fasting and non-fasting states, enhancing its utility in real-world clinical assessments (18). Third, its broad applicability has been demonstrated across various metabolic and vascular conditions—including cardiovascular disease (21), thyroid dysfunction (22), depression (23), kidney stones (24), suicidal ideation (25), osteoporosis (26), and even abdominal aortic aneurysm (AAA) (27).

Given that PAD is essentially an atherosclerotic disease, a higher NHHR—reflecting an imbalance between atherogenic and protective lipoproteins—may contribute to endothelial dysfunction, inflammation, and lipid deposition in peripheral arteries (28), thereby increasing the risk of PAD. Previous studies have indicated that in patients with PAD, the NHHR has a U-shaped relationship with major adverse cardiovascular events (29). These findings suggest that dynamic monitoring of the NHHR may aid in risk stratification and personalized lipid management for PAD patients. Furthermore, owing to variations in genetic, environmental, and lifestyle factors, the risk factors for and disease progression of PAD may differ among populations. As the incidence and clinical burden of PAD increase sharply with advancing age, individuals aged ≥50 years represent the population at highest risk and constitute the main demographic group undergoing vascular evaluation and surgical intervention (30, 31). Focusing on this group ensures clinical relevance and adequate event rates while minimizing the heterogeneity related to age-dependent metabolic variation. Therefore, the present study aimed to clarify the relationship between the NHHR and PAD in a Chinese inpatient population aged ≥50 years undergoing vascular surgery to inform the early clinical assessment and management of PAD.

## Materials and methods

### Ethical considerations

The study protocol was approved by the Ethics Committee of the Affiliated Kunshan Hospital of Jiangsu University, Suzhou, China (Approval No. 2025-03-001-H00-KOl), and the research was conducted in accordance with the Declaration of Helsinki. All patient data were anonymized to ensure confidentiality, and written informed consent was obtained from each participant before inclusion.

### Study design and setting

This was a retrospective cross-sectional study involving inpatients aged ≥50 years who underwent vascular surgery at Affiliated Kunshan Hospital of Jiangsu University between December 2017 and August 2024. The data were retrospectively collected from the medical records of patients during this timeframe.

### Participants

**Figure 1:**
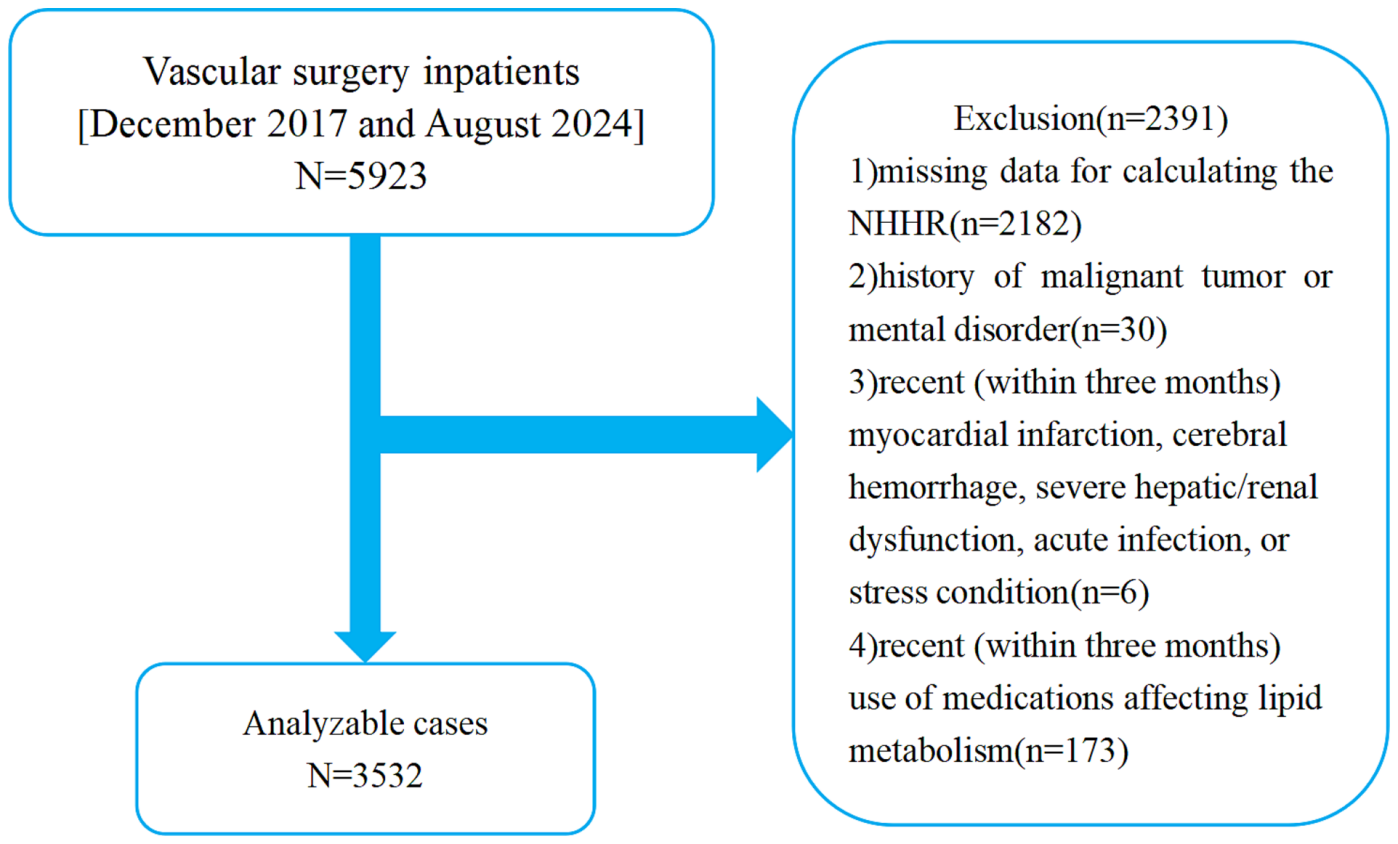
Diagrammatic representation of the study design. NHHR, Non-high-density lipoprotein cholesterol to high-density lipoprotein cholesterol ratio.

Initially, 5,923 patients were identified. After the medical records were retrieved and the documentation of routine blood test results was ensured, the patients were screened according to predefined criteria. The inclusion criteria were as follows: (1) age ≥50 years and (2) complete clinical and biochemical data (including total cholesterol and HDL-C levels). The exclusion criteria were as follows: (1) missing data for calculating the NHHR; (2) a history of malignant tumours or mental disorders; (3) recent (within three months) myocardial infarction, cerebral haemorrhage, severe hepatic/renal dysfunction, acute infection, or stress conditions; and (4) recent (within three months) use of medications affecting lipid metabolism (27). These exclusion criteria were implemented primarily to minimize potential confounding factors that could affect lipid metabolism. Specifically, individuals with recent acute medical conditions or those with a history of significant health issues may experience alterations in lipid profiles, which could distort the results of the analysis. This approach ensures a more accurate evaluation of the NHHR in the context of PAD risk. However, this may also limit the external validity of the findings, as the selected cohort may not fully represent the broader population of patients with PAD. The application of these criteria led to the exclusion of 2,391 patients, resulting in the enrolment of 3,532 participants in the analysis (Figure 1).

### Variables and measurement

The exposure variable in this study was the NHHR. The NHHR was calculated using the formula:


$$\text{NHHR}=\frac{\text{non}-\mathrm{HDL}-C}{\mathrm{HDL}-C\;}$$

Where non-HDL-C was determined by subtracting the HDL-C level from the TC level. Serum TC was measured using an enzymatic colorimetric method based on the cholesterol oxidase–peroxidase (CHOD–POD) principle, and HDL-C was determined using a homogeneous enzymatic method, both of which were performed on a Beckman AU5800 automatic biochemical analyser (Beckman Coulter, Brea, CA, USA).

The outcome variable was the presence of PAD, defined by characteristic symptoms and an abnormal ankle brachial index (ABI). Patients were initially assessed for PAD-like symptoms according to criteria established by the Task Force of the Society for Vascular Surgery and the International Society for Cardiovascular Surgery (31, 32), and the ABI was measured by experienced clinicians using standard protocols. PAD was defined as the presence of PAD-like symptoms with an ABI < 0.9 (33).

The covariates collected in this study included age, sex, lipoprotein(a) level (Lp[a]), apolipoprotein A1 level (Apo A1), alanine aminotransferase level (ALT), neutrophil count (NEUT), hypertension status, diabetes status, smoking status, and alcohol consumption status. Lp(a) and Apo A1 levels were measured by immunoassay, and ALT levels were measured by an enzymatic assay using a Beckman AU5800 autoanalyser. NEUT was determined using flow cytometry with a Sysmex XN-10 analyser (Kobe, Hyogo, Japan). Hypertension and diabetes were defined as cases previously diagnosed by a medical institution. Smoking was defined as smoking at least one cigarette daily in the past 30 days (34). Drinking was defined as the consumption of alcohol at least once per week during the past 6 months (35). All clinical and biochemical parameters were evaluated in the fasting state within three days of patient admission to ensure consistency across measurements.

### Study size

We performed *a priori* power analysis using GPower to assess sample size adequacy, with standard parameters (*α* = 0.05, power = 0.80). For the primary analysis, targeting an odds ratio (OR) of 0.77, the calculated required sample size was 3,234, whereas our actual sample size was 3,532, ensuring adequate statistical power. However, in certain smaller subgroups, the sample sizes may be insufficient to meet the a priori power requirements, potentially affecting the robustness of the subgroup analyses.

### Statistical methods

Continuous variables were tested for normality using the Shapiro**–**Wilk test. Categorical variables are expressed as counts (percentages), whereas continuous variables are shown as the mean ± standard deviation (SD) if normally distributed or as the median (Q1–Q3) if nonnormally distributed. For univariate comparisons, categorical data were analysed by Pearson chi-square or Fisher’s exact tests. Continuous data were compared using independent-samples t tests for normally distributed data or Mann–Whitney U tests for nonnormally distributed data.

Univariate analyses were first performed to explore the associations between PAD and patient characteristics. Following this, standard logistic regression was used to examine the potential independent association between the NHHR and the prevalence of PAD. Three models were constructed, namely, Model 1 (unadjusted), Model 2 (adjusted for key covariates), and Model 3 (fully adjusted for all covariates). Variance inflation factors (VIFs) were calculated to check for multicollinearity. Covariates were retained in the final model if either (1) the NHHR–PAD odds ratio (OR) changed by ≥10% when the covariate was added or removed or (2) the covariate had a *p* value ≤0.10 in univariate analysis or Model 1.

Notably, the variables “smoking” and “alcohol consumption” had some missing values. To handle these missing data, a separate “unknown” category was created for these variables so that all patients could be included in the multivariate models without bias. This approach enabled the retention of these covariates in the adjusted analysis without compromising the validity of the model. Additionally, missing NHHR values were attributed mainly to manual data omission. To assess the potential influence of the missing data on our results, we summarized the baseline characteristics of the excluded patients and compared them with those of the analysed cohort to evaluate possible selection bias. Furthermore, sensitivity analyses were conducted using random forest-based multiple imputation to verify the robustness of the findings.

The study population was divided into subgroups stratified by relevant covariates to assess the consistency of the NHHR–PAD association across different patient subsets. Interaction effects between the NHHR and subgroup factors were evaluated using likelihood ratio tests (LRTs). A generalized additive model (GAM) was also used to examine potential nonlinear relationships. If a nonlinear association was suggested, piecewise logistic regression was applied to estimate the threshold effect point on the smoothed curve. If there were differences in slope between segments of the curve, the inflection point was recursively determined using maximum likelihood estimation.

All analyses were performed using EmpowerStats (X&Y Solutions, MA, USA) and R software (R Foundation for Statistical Computing, Vienna, Austria). A two-sided *p* value < 0.05 was considered to indicate statistical significance.

## Results

### Clinical and demographic characteristics of the study participants

A total of 3,532 inpatients aged ≥50 years who underwent vascular surgery between December 2017 and August 2024 were included in the analysis. The baseline characteristics of the participants in terms of NHHR quartile (Q1–Q4) are presented in Table 1. The mean age of the patients was 66.3 ± 9.1 years, and 55.8% were male. The overall prevalence of PAD was 8.89%, and the mean NHHR was 2.30 ± 0.76. Significant differences in various variables were observed among the NHHR quartiles, specifically, Lp(a), Apo A1, ALT, NEUT, total cholesterol, and HDL-C (*p* ≤ 0.05 for each).

**Table 1:** Patient characteristics based on NHHR quartiles.

	Mean ± SD					
NHHR	Q1	Q2	Q3	Q4	*p*-value	*p*-value*
*N*	883	883	883	883		
NHHR	1.43 ± 0.25	1.99 ± 0.13	2.46 ± 0.15	3.33 ± 0.54	<0.001	<0.001
Age, years	68.48 ± 9.65	66.36 ± 8.98	65.49 ± 8.73	64.82 ± 8.77	<0.001	<0.001
Apo A1, g/L	1.45 ± 0.31	1.37 ± 0.27	1.30 ± 0.24	1.20 ± 0.22	<0.001	<0.001
NEUT, 10^9/L	3.72 ± 1.90	3.69 ± 1.60	3.70 ± 1.45	3.89 ± 1.85	0.042	<0.001
TC, mmol/L	3.84 ± 0.84	4.38 ± 0.86	4.76 ± 0.92	5.21 ± 0.99	<0.001	<0.001
HDL-C, mmol/L	1.59 ± 0.34	1.46 ± 0.29	1.38 ± 0.26	1.21 ± 0.24	<0.001	<0.001
Median (Q1–Q3)						
Lp(a), mg/L	118.00 (64.00–239.50)	141.00 (77.00–257.00)	151.00 (74.00–285.50)	140.00 (74.00–288.00)	<0.001	<0.001
ALT, U/L	18.00 (14.00–25.00)	18.00 (14.00–25.00)	19.00 (14.00–26.00)	20.00 (15.00–28.00)	<0.001	<0.001
*N* (%)						
Sex, *N* (%)					0.008	-
Female	358 (40.54%)	415 (47.00%)	415 (47.00%)	373 (42.24%)		
Male	525 (59.46%)	468 (53.00%)	468 (53.00%)	510 (57.76%)		
Smoking, *N* (%)					0.194	-
No	626 (70.89%)	602 (68.18%)	608 (68.86%)	577 (65.35%)		
Yes	77 (8.72%)	74 (8.38%)	68 (7.70%)	88 (9.97%)		
Unknown	180 (20.39%)	207 (23.44%)	207 (23.44%)	218 (24.69%)		
Drinking, *N* (%)					0.068	-
No	639 (72.37%)	636 (72.03%)	633 (71.69%)	621 (70.33%)		
Yes	64 (7.25%)	40 (4.53%)	43 (4.87%)	44 (4.98%)		
Unknown	180 (20.39%)	207 (23.44%)	207 (23.44%)	218 (24.69%)		
Hypertension, *N* (%)					0.001	-
No	536 (60.70%)	567 (64.21%)	594 (67.27%)	521 (59.00%)		
Yes	347 (39.30%)	316 (35.79%)	289 (32.73%)	362 (41.00%)		
Diabetes, *N* (%)					0.001	-
No	805 (91.17%)	806 (91.28%)	797 (90.26%)	762 (86.30%)		
Yes	78 (8.83%)	77 (8.72%)	86 (9.74%)	121 (13.70%)		
PAD, %					<0.001	-
No	775 (87.77%)	807 (91.39%)	826 (93.54%)	810 (91.73%)		
Yes	108 (12.23%)	76 (8.61%)	57 (6.46%)	73 (8.27%)		

NHHR, non-high-density lipoprotein cholesterol to high-density lipoprotein cholesterol ratio; SD, standard deviation; Q1, first quartile; Q2, second quartile; Q3, third quartile; Q4, fourth quartile; Apo A1, apolipoprotein A1; NEUT, neutrophil count; TC, total cholesterol; HDL-C, high-density lipoprotein cholesterol; Lp(a), lipoprotein(a); ALT, alanine aminotransferase; PAD, peripheral artery disease.

*p*-value*: Kruskal Wallis rank test for continuous variables, Fisher exact for categorical variables with expects<10.

### Univariate analysis of PAD risk factors

Univariate analysis was used to examine the relationships between the various demographic, clinical, and biochemical variables with PAD. Variables that were found to be significantly associated with PAD included age, sex, Lp(a), Apo A1, NEUT, TC, HDL-C, the NHHR, hypertension, diabetes, and smoking (Supplementary Table 1). No significant correlations were observed for ALT or alcohol consumption in the univariate analysis.

### Associations between the NHHR and PAD (multivariate analysis)

To assess multicollinearity among the variables, variance inflation factors (VIFs) were calculated for each variable included in the logistic regression analysis (Supplementary Table 2). The results of the multivariate logistic regression analysis (Table 2) consistently revealed an inverse association between the NHHR and PAD after adjustment for confounders. According to the fully adjusted model (Model 3), the NHHR was significantly associated with a lower PAD risk (OR 0.77, 95% CI 0.65–0.93; *p* = 0.006). When the NHHR was analysed by quartile, the third quartile (Q3) had the lowest PAD risk in all the models. When Q1 was used as the reference in the fully adjusted model, the adjusted ORs for PAD were 0.72 (95% CI 0.51–1.02; *p* = 0.065) for Q2, 0.51 (95% CI 0.35–0.75; *p* < 0.001) for Q3, and 0.54 (95% CI 0.37–0.79; *p* = 0.002) for Q4. Thus, patients in the higher NHHR quartiles (especially Q3 and Q4) had a significantly lower likelihood of developing PAD than those in Q1 did.

**Table 2:** Association between NHHR and PAD in different models.

NHHR	Cases/n	Model 1^a^	Model 2^b^	Model 3^c^			
		OR(95%CI)	*P*-value	OR (95%CI)	*P*-value	OR (95%CI)	*P*-value
Per 1 SD (0.60)	314/3532	0.83 (0.71, 0.98)	0.023	0.89 (0.76, 1.05)	0.170	0.77 (0.65, 0.93)	0.006
Q1	108/883	1.0		1.0		1.0	
Q2	76/883	0.68 (0.50, 0.92)	0.013	0.82 (0.58, 1.16)	0.258	0.72 (0.51, 1.02)	0.065
Q3	57/883	0.50 (0.35, 0.69)	<0.001	0.63 (0.44, 0.91)	0.014	0.51 (0.35, 0.75)	<0.001
Q4	73/883	0.65 (0.47, 0.88)	0.006	0.74 (0.52, 1.05)	0.092	0.54 (0.37, 0.79)	0.002

^a^No adjustment.

^b^Adjusted for age, sex, hypertension, diabetes.

^c^Adjusted for age, sex, Lp(a), Apo A1, ALT, NEUT, smoking, drinking, hypertension, diabetes.

NHHR, non-high-density lipoprotein cholesterol to high-density lipoprotein cholesterol ratio; PAD, peripheral artery disease; Lp(a), lipoprotein(a); Apo A1, apolipoprotein A1; ALT, alanine aminotransferase; NEUT, neutrophil count.

### Sensitivity analysis with multiple imputations for missing data

The baseline characteristics of the included participants (*N* = 3,532) and those who were excluded (*N* = 2,391) are summarized in Supplementary Table 3. Significant differences were observed between the two groups in terms of age; the prevalence of PAD, ALT, and NEUT; and comorbidities, including hypertension, diabetes, smoking, and alcohol consumption. However, no significant differences in key metabolic parameters, such as Lp(a), Apo A1, or sex distribution, were detected. The number of PAD cases among the excluded patients is listed in Supplementary Table 4. A total of 257 PAD patients were excluded for the following reasons: 225 patients were excluded because of missing NHHR data; 4 patients had a history of malignancy or mental disorders; 1 patient had recently experienced myocardial infarction, stroke, or severe organ dysfunction; and 27 patients had recently used lipid-lowering medications.

Sensitivity analyses using random forest multiple imputation for missing NHHR values, as well as for missing smoking and drinking data, demonstrated results consistent with those of the primary analysis (Supplementary Tables 5, 6).

### Spline analysis and threshold effects

**Figure 2:**
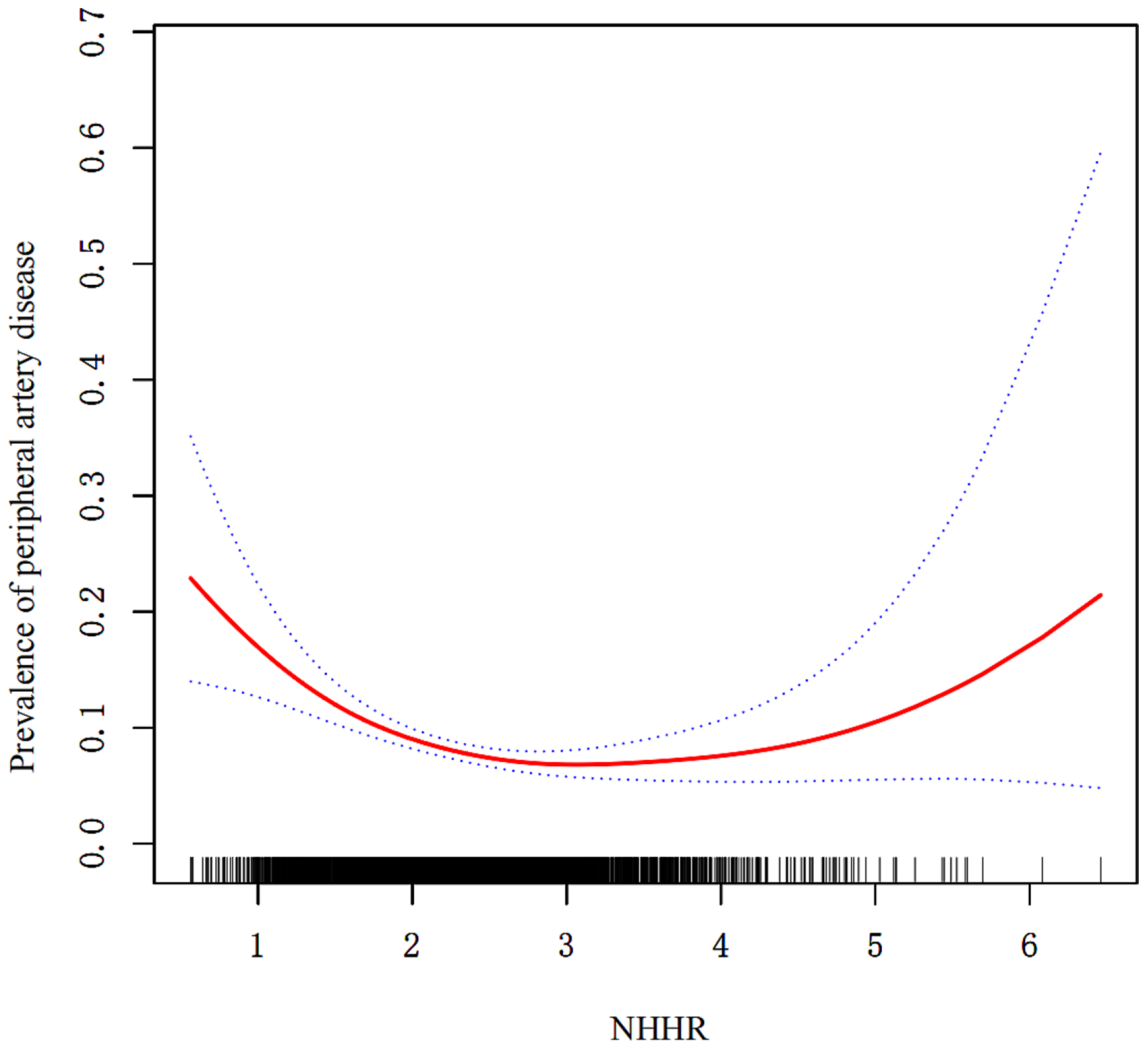
Smoothed adjusted curves illustrating the relationship between the NHHR and PAD. The red line indicates the nonlinear association between the NHHR and PAD, while the blue line represents the 95% CI. The nonlinear correlation was maintained after controlling for variables such as age, sex, Lp(a), Apo A1, ALT, NEUT, hypertension, diabetes, smoking status, and alcohol consumption.

Adjusted GAM spline analysis (Table 3; Figure 2) was used to explore nonlinear patterns in the NHHR–PAD relationship. After controlling for age, sex, Lp(a) level, Apo A1 level, ALT level, NEUT, smoking status, alcohol consumption status, hypertension status, and diabetes status, the NHHR–PAD association was observed to be significantly nonlinear. An inflection point was identified at an NHHR value of approximately 2.75. Below this value, higher NHHR values were associated with a markedly lower risk of PAD (OR 0.51, 95% CI 0.38–0.67; *p* < 0.001), whereas above this threshold, the reverse situation was observed, with higher NHHR values linked to an increased PAD risk (OR 1.44, 95% CI 1.03–2.00; *p* = 0.033). These results indicated the presence of a “J-shaped” relationship between the NHHR and PAD risk, wherein the NHHR was protective up to a threshold (approximately NHHR = 2.75) but had potentially deleterious effects at values above this threshold.

**Table 3:** Threshold analyses examining the relationship between NHHR and PAD.

Model	Model 3^a^
	OR (95%CI) *p*-value
Model A^b^	
One line slope	0.77 (0.65, 0.93) 0.006
Model B^c^	
NHHR turning point (K)	2.75
< K	0.51 (0.38, 0.67) < 0.001
> K	1.44 (1.03, 2.00) 0.033
Slope 2-Slope 1	2.84 (1.71, 4.73) < 0.001
LRT^d^	<0.001

^a^Adjusted for age, sex, Lp(a), Apo A1, ALT, NEUT, smoking, drinking, hypertension, diabetes.

^b^Linear analysis, *p* value<0.05 indicates a linear relationship.

^c^Nonlinear analysis.

^d^*P* value<0.05 means Model B is significantly different from Model A, which indicates a nonlinear relationship.

### Subgroup analyses

The NHHR–PAD association was examined within subgroups defined by age, sex, smoking status, alcohol consumption status, hypertension status, diabetes status, Lp(a) level, Apo A1 level, ALT level, and NEUT (Table 4), with adjustments for other covariates. Overall, the inverse association between the NHHR and PAD persisted across the subgroups. However, significant interaction effects were noted for sex (interaction *p* < 0.05). Specifically, the NHHR–PAD association was attenuated and nonsignificant in male patients (OR 0.90, 95% CI 0.73–1.10; *p* = 0.298), whereas in female patients, the NHHR remained strongly protective against PAD (OR 0.53, 95% CI 0.38–0.75; *p* < 0.001).

**Table 4:** Subgroup analyses examining the relationship between NHHR and PAD.

Subgroup	*N*	OR (95% CI) *P*-value	Interaction *P*-value
Age, years			
>50, ≤70	2,411	0.82 (0.62, 1.07) 0.139	0.263
>70	1,121	0.67 (0.54, 0.84) < 0.001	
Sex			
Female	1,561	0.53 (0.38, 0.75) < 0.001	0.007
Male	1971	0.90 (0.73, 1.10) 0.298	
Smoking			
No	2,413	0.75 (0.60, 0.95) 0.016	0.335
Yes	307	1.03 (0.67, 1.58) 0.888	
Unknown	812	0.69 (0.49, 0.98) 0.039	
Drinking			
No	2,529	0.78 (0.63,0.96) 0.021	0.321
Yes	191	1.33 (0.62,2.85) 0.467	
Unknown	812	0.69 (0.49,0.98) 0.040	
Hypertension			
No	2,218	0.63 (0.45, 0.87) 0.005	0.121
Yes	1,314	0.84 (0.68, 1.03) 0.101	
Diabetes			
No	3,170	0.70 (0.56, 0.88) 0.002	0.150
Yes	362	0.91 (0.69, 1.21) 0.521	
Lp(a), mg/L			
≤300	2,782	0.84 (0.69, 1.03) 0.093	0.071
>300	750	0.58 (0.40, 0.85) 0.005	
Apo A1, g/L			
≤1.35	2053	0.83 (0.68, 1.02) 0.071	0.847
>1.35	1,479	0.80 (0.56, 1.13) 0.208	
NEUT, 10^9/L			
≤6.0	3,301	0.79 (0.65, 0.95) 0.013	0.608
>6.0	231	0.68 (0.41, 1.15) 0.152	
ALT, U/L			
≤40.0	3,225	0.79 (0.66, 0.95) 0.015	0.319
>40.0	307	0.57 (0.31, 1.08) 0.086	

Patients were stratified based on age, sex, smoking status, alcohol consumption, hypertension, diabetes, Lp(a), Apo A1, NEUT, and ALT, and additional covariates not included in the stratification were adjusted for in the analysis.

NHHR, non-high-density lipoprotein cholesterol to high-density lipoprotein cholesterol ratio; PAD, peripheral artery disease; Lp(a), lipoprotein(a); Apo A1, apolipoprotein A1; NEUT, neutrophil count; ALT, alanine aminotransferase.

## Discussion

The findings of this cross-sectional analysis of 3,532 inpatients aged ≥50 years who underwent vascular surgery indicated that the NHHR was inversely associated with the prevalence of PAD after controlling for confounders. Patients in the third NHHR quartile were found to have the lowest PAD risk. The relationship between the NHHR and PAD was nonlinear and J shaped, with an inflection of approximately NHHR = 2.75. NHHR values below this threshold were protective against PAD, whereas those above 2.75 were associated with a higher PAD risk. Moreover, this association was significantly modified by sex and was more pronounced among women. These results indicate that the risk of PAD in relation to the NHHR is affected by patient subgroup (e.g., patient sex), underscoring the need for personalized risk assessment and management strategies.

The present findings align with prior evidence linking dyslipidaemia to PAD risk in the lower extremities. For example, Bertrand et al. (17) reported that higher HDL-C levels were associated with lower PAD prevalence and incidence in patients with diabetes, whereas higher TC/HDL-C ratios and non-HDL-C levels were associated with a greater risk of PAD. Similarly, Liu et al. (14) observed in a community-based study that cumulative exposure to elevated LDL-C levels was an independent risk factor for PAD development, whereas non-HDL-C levels had no significant predictive value. In a large study of hypertensive adults, Ding et al. noted that several composite lipid indices (TC/HDL-C, TG/HDL-C, LDL-C/HDL-C, and non-HDL-C) were independently and positively correlated with PAD, with the TC/HDL-C and LDL-C/HDL-C ratios being the most strongly predictive (16). These studies confirm the importance of abnormal lipid profiles in PAD pathogenesis and emphasize the importance of aggressive lipid management for the prevention of PAD in at-risk patients.

In contrast to earlier studies conducted in general or diabetic populations, the present analysis focused specifically on an older Chinese inpatient cohort at high risk for PAD, evaluating the use of the NHHR, a novel composite lipid marker that integrates both atherogenic (non-HDL-C) and protective (HDL-C) cholesterol components, for a more holistic assessment of PAD risk. Notably, a clear threshold effect was identified in the NHHR–PAD relationship, and this relationship was modified by patient sex, findings that have not been reported previously. These results contribute to the literature by demonstrating that the NHHR is associated with PAD prevalence in an older vascular surgical patient population while also emphasizing the importance of considering patient sex when evaluating the NHHR for PAD risk stratification.

The NHHR may influence the prevalence of PAD through various mechanisms. Disturbances in glucose and lipid metabolism are important contributors to PAD pathogenesis. Insulin resistance can reduce the activity of lipoprotein lipase, impair catabolism of very-low-density lipoprotein (VLDL), and promote hepatic production of apoB100-containing lipoproteins, thereby increasing plasma triglyceride (TG) and remnant cholesterol (RC) levels (36). An elevated NHHR indicates an atherogenic, “lipotoxic” state characterized by endoplasmic reticulum stress, mitochondrial dysfunction, and oxidative stress, which can aggravate insulin resistance and pancreatic *β*-cell dysfunction (37, 38). Moreover, an increased TC: HDL-C ratio is associated with increased PAD risk, suggesting that imbalances in lipid ratios contribute to the formation of atherosclerotic plaques (14, 16). Conversely, extremely elevated HDL-C levels may reflect impaired reverse cholesterol transport or HDL dysfunction, potentially promoting cholesterol deposition in vascular tissues and contributing to atherogenesis (19, 39). This paradoxical association between very high HDL-C levels and adverse outcomes might partially explain the J-shaped relationship observed between the NHHR and PAD risk in the present study, suggesting that both very low and very high NHHR values could be linked to increased risk.

An elevated NHHR reflects a proinflammatory milieu. Small, dense LDL particles are readily oxidized to oxLDL, which is subsequently taken up by macrophages *via* scavenger receptors, resulting in the formation of foam cells. These foam cells secrete inflammatory cytokines (e.g., IL-6 and TNF-*α*), exacerbating vascular inflammation and atherosclerosis (36, 40). Clinically, higher levels of non-HDL-C are correlated with increased activity of proinflammatory macrophages, especially in obesity (41). Thus, an elevated NHHR likely perpetuates inflammation by increasing the levels of oxidized lipids and activating macrophage-driven inflammatory responses, accelerating PAD progression.

Endocrine factors exert a substantial influence on PAD risk partly through their regulation of lipid and glucose metabolism. For instance, insulin deficiency or resistance can downregulate hepatic LDL receptor expression and impair LDL clearance while simultaneously increasing hormone-sensitive lipase activity, leading to elevated free fatty acid levels and atherogenic dyslipidaemia (36). In addition, sex hormones may account for the sex-specific differences observed in the NHHR–PAD association. Previous studies have shown that higher HDL-C concentrations do not always confer cardiovascular benefit in women and, in some cases, are even associated with increased coronary risk (42). These paradoxical findings are attributed to HDL functional heterogeneity—in which HDL particles may become dysfunctional under oestrogen deficiency, oxidative stress, or chronic inflammation. In women, particularly those in the perimenopausal or postmenopausal stages, the withdrawal of oestrogen alters HDL composition and impairs ApoA1-mediated cholesterol efflux and antioxidative activity. Consequently, despite higher HDL-C levels, the overall functionality of HDL may decline, reducing its vascular protective effects (42). This decline in HDL functionality may explain why the association between the NHHR and PAD appears stronger in women. Therefore, future investigations should focus on the quality of HDL rather than solely its quantity, particularly in the context of menopause, to better understand the underlying mechanisms affecting cardiovascular risk in women.

Moreover, a high NHHR may also harm endothelial cells through oxidative mechanisms, thereby promoting the occurrence of PAD. Oxidized LDL impairs the function of endothelial nitric oxide synthase (eNOS), lowering nitric oxide availability and promoting vasoconstriction and platelet aggregation (36). OxLDL also triggers the apoptosis of endothelial cells, increases vascular permeability, and promotes monocyte adhesion and migration, accelerating plaque development (36, 43). Moreover, imbalances in lipid ratios (e.g., high LDL-C/HDL-C) can activate the NF-κB pathway in endothelial cells, exacerbating local inflammation (16). Thus, it is likely that an elevated NHHR contributes to PAD by inducing endothelial dysfunction and inflammation in arterial walls.

Additionally, genetic susceptibility may modulate the influence of dyslipidaemia on the risk of PAD. For example, the apolipoprotein Eε4 allele is linked to increased susceptibility to PAD development, likely because of alterations in lipoprotein metabolism and increased inflammation (44). Similarly, mutations in genes associated with familial hypercholesterolemia (e.g., LDLR and PCSK9) result in lifelong elevated levels of LDL-C, which accelerate atherosclerosis and PAD development (14). These genetic factors may partly explain interindividual variability in PAD risk despite the presence of similar lipid profiles, suggesting a role for genetic screening in high-risk patients.

The findings of the present study have significant clinical implications for assessing the risk of PAD. The observed nonlinear (J-shaped) relationship between the NHHR and PAD suggests the need for a nuanced and personalized approach. Specifically, NHHR values above approximately 2.75 were associated with an increasing risk of PAD, while values well below this threshold were also linked to a higher PAD risk. This finding indicates that while a low non-HDL-C/high HDL-C profile is generally viewed as favourable, excessively low NHHR values may paradoxically be detrimental to the health of peripheral arteries.

Clinicians should be mindful of this threshold effect, recognizing that patients with either very low or very high NHHR levels may both require closer monitoring, rather than assuming that “lower is always better” in terms of the NHHR. Regular monitoring of the NHHR may provide useful information for clinicians assessing PAD risk in patients undergoing vascular surgery. Identifying patients at the extremes of the NHHR spectrum may facilitate early preventive interventions—such as tailored lipid-modifying therapy and optimization of metabolic profiles—aimed at mitigating PAD risk and promoting vascular health. Additionally, sex interaction effects in the context of NHHR and PAD risk were explored in this study. Preliminary analyses suggest that the relationship between the NHHR and PAD may differ by sex, highlighting the need for further investigations to replicate these findings and confirm their clinical relevance.

A key strength of the present study is its use of three analytic models (unadjusted, partially adjusted, and fully adjusted) to assess the robustness of the association between the NHHR and PAD, thereby minimizing potential confounding bias. Additionally, unlike the ABI, which requires specialized equipment and training to measure, the NHHR can be readily determined from routine bloodwork. These findings suggest that the NHHR may be a potentially useful marker for identifying patients who warrant further evaluation for PAD risk, but validation through prospective studies is needed to confirm its clinical utility.

Nevertheless, this study has several limitations. First, its cross-sectional design limits causal inference, and it cannot be determined whether an elevated NHHR causes PAD or is merely associated with it. Prospective studies are needed to clarify causality. Second, 2,391 patients were excluded primarily because of incomplete data, along with a small number of patients who had recent acute illnesses or significant health issues. While multiple imputation analyses supported the robustness of our findings, the potential influence of missing data and these excluded conditions on the results cannot be completely ruled out. Third, patients receiving lipid-modifying medications were excluded. This exclusion was primarily due to our inability to obtain detailed medication data on the use of drugs that affect lipid metabolism. Since the use of such medications can substantially influence the outcomes of lipid profiles and related analyses, we opted to exclude these patients to maintain the integrity of our results. While this improved internal validity by isolating the effect of the NHHR, it may have reduced external validity, as many real-world patients are on such therapies. Excluding patients receiving lipid-modifying therapy may limit the applicability of our results to real-world patients who are on standard lipid management. Therefore, future studies should consider a more comprehensive patient population, such as one including medication history, to increase the generalizability of the findings. Fourth, the sample size (*n* = 3,532) and single-centre nature may limit the generalizability of the results and the statistical power for subgroup analyses. Additionally, the data were collected from inpatients aged ≥50 years who underwent vascular surgery at a single tertiary hospital in China, which may introduce clustering effects related to the performing surgeon, surgical techniques, and institutional practices. Larger multicentre studies involving more diverse populations are warranted to confirm these findings. Fifth, while we have discussed the association between the NHHR and PAD, the possibility of reverse causation cannot be completely ruled out; specifically, the presence of PAD may influence lipid metabolism and subsequently affect the NHHR. Addressing these issues in future research will provide clearer insights into the relationship between the NHHR and PAD, helping to differentiate the causal pathways and interactions involved. Sixth, although we adjusted for several important covariates, unmeasured confounding factors such as body mass index, renal function, sex hormones, inflammatory markers, dietary habits, physical activity, genetic predisposition, and potential misclassification of PAD may still influence the observed associations. Therefore, future studies that encompass a broad range of lifestyle factors, biological markers, and genetic data are essential for further understanding the role of the NHHR in PAD. Finally, while a nonlinear association between the NHHR and PAD, as well as a potential sex-related interaction, was identified, these findings are exploratory in nature. The underlying biological mechanisms require further investigation, highlighting the need for additional mechanistic and prospective studies.

## Conclusion

This study revealed an association between the NHHR and the presence of peripheral artery disease, with evidence suggesting a nonlinear pattern and potential sex-specific differences. Importantly, owing to the retrospective cross-sectional design, these findings do not support causal inference or strong predictive claims. These results indicate that this biomarker may help identify individuals who could benefit from further clinical evaluation rather than serve as a definitive screening or predictive tool. Future prospective studies are necessary to clarify the temporal relationship and to determine its clinical value in risk assessment and decision-making.

## Data Availability

The raw data supporting the conclusions of this article will be made available by the authors, without undue reservation.
