# Supplementary material for: Association between the non-high-density lipoprotein cholesterol to high-density lipoprotein cholesterol ratio and peripheral artery disease in vascular surgery inpatients aged 50 and above: a retrospective cross-sectional study
Source: Front Med (Lausanne). 2026 Jan 21;13:1739515. doi: 10.3389/fmed.2026.1739515 (PMC12868209; doi:10.3389/fmed.2026.1739515)
Supplement: Supplementary file 1 [file Table_1.docx]

Supplementary Table 1. Univariate analysis of associated risk factors for PAD.

| Characteristics | Statistics | OR (95%CI) P-value |
| --- | --- | --- |
| Age, years | 66.29 ± 9.14 | 1.11 (1.10, 1.13) <0.001 |
| Apo A1, g/L | 1.33 ± 0.28 | 0.13 (0.08, 0.21) <0.001 |
| NEUT, 10^9/L | 3.75 ± 1.71 | 1.23 (1.17, 1.30) <0.001 |
| TC, mmol/L | 4.54 ± 1.03 | 0.50 (0.44, 0.57) <0.001 |
| HDL-C, mmol/L | 1.41 ± 0.32 | 0.16 (0.11, 0.24) <0.001 |
| NHHR | 2.30 ± 0.76 | 0.83 (0.71, 0.98) 0.023 |
| Median (Q1–Q3) |  |  |
| Lp(a), mg/L | 137.00 (72.00-268.00) | 1.00 (1.00, 1.00) <0.001 |
| ALT, U/L | 19.00 (14.00-26.00) | 0.99 (0.99, 1.00) 0.128 |
| N (%) |  |  |
| Sex, N (%) |  |  |
| Female | 1561 (44.20%) | 0.40 (0.31, 0.52) <0.001 |
| Male | 1971 (55.80%) | Reference |
| Smoking, N (%) |  |  |
| No | 2413 (68.32%) | Reference |
| Yes | 307 (8.69%) | 2.28 (1.62, 3.19) <0.001 |
| Unknown | 812 (22.99%) | 1.17 (0.89, 1.55) 0.266 |
| Drinking, N (%) |  |  |
| No | 2529 (71.60%) | Reference |
| Yes | 191 (5.41%) | 0.74 (0.41, 1.32) 0.310 |
| Unknown | 812 (22.99%) | 1.02 (0.77, 1.34) 0.905 |
| Hypertension, N (%) |  |  |
| No | 2218 (62.80%) | Reference |
| Yes | 1314 (37.20%) | 4.47 (3.48, 5.75) <0.001 |
| Diabetes, N (%) |  |  |
| No | 3170 (89.75%) | Reference |
| Yes | 362 (10.25%) | 6.01 (4.60, 7.84) <0.001 |

PAD, peripheral artery disease; Apo A1, apolipoprotein A1; NEUT, neutrophil count; TC, total cholesterol; HDL-C, high-density lipoprotein cholesterol; NHHR, non-high-density lipoprotein cholesterol to high-density lipoprotein cholesterol ratio; Lp(a), lipoprotein(a); ALT, alanine aminotransferase.
