# Supplementary material for: Association between the non-high-density lipoprotein cholesterol to high-density lipoprotein cholesterol ratio and peripheral artery disease in vascular surgery inpatients aged 50 and above: a retrospective cross-sectional study
Source: Front Med (Lausanne). 2026 Jan 21;13:1739515. doi: 10.3389/fmed.2026.1739515 (PMC12868209; doi:10.3389/fmed.2026.1739515)
Supplement: Supplementary file 2 [file Table_2.docx]

Supplementary Table 2. Variance inflation factor values for variables in the logistic regression analysis.

| Variables | Variance inflation factor |
| --- | --- |
| Age, years | 1.2 |
| Apo A1, g/L | 1.3 |
| NEUT, 10^9/L | 1.1 |
| Lp(a), mg/L | 1.0 |
| ALT, U/L | 1.0 |
| Sex, N (%) | 1.2 |
| Smoking, N (%) | 1.4 |
| Drinking, N (%) | 1.3 |
| Hypertension, N (%) | 1.1 |
| Diabetes, N (%) | 1.1 |

Apo A1, apolipoprotein A1; NEUT, neutrophil count; Lp(a), lipoprotein(a); ALT, alanine aminotransferase.
