# Supplementary material for: Association between the non-high-density lipoprotein cholesterol to high-density lipoprotein cholesterol ratio and peripheral artery disease in vascular surgery inpatients aged 50 and above: a retrospective cross-sectional study
Source: Front Med (Lausanne). 2026 Jan 21;13:1739515. doi: 10.3389/fmed.2026.1739515 (PMC12868209; doi:10.3389/fmed.2026.1739515)
Supplement: Supplementary file 3 [file Table_3.docx]

Supplementary Table 3. Patient characteristics based on inclusion and exclusion for the study.

|  | Mean±SD / N (%) | |  |  |
| --- | --- | --- | --- | --- |
|  | Excluded patients | Include patients | P-value | P-value* |
| N | 2391 | 3532 |  |  |
| NHHR | - | 2.30±0.76 | - | - |
| Age, years | 68.48±9.97 | 66.29±9.14 | <0.001 | <0.001 |
| Apo A1, g/L | 1.16±0.36 | 1.33±0.28 | 0.006 | 0.101 |
| NEUT, 10^9/L | 5.13±3.25 | 3.75±1.71 | <0.001 | <0.001 |
| TC, mmol/L | - | 4.54±1.03 | - | - |
| HDL-C, mmol/L | - | 1.41±0.32 | - | - |
| Median (Q1–Q3) |  |  |  |  |
| Lp(a), mg/L | 83.00 (32.00-232.00) | 137.00 (72.00-268.00) | 0.367 | 0.104 |
| ALT, U/L | 20.00 (15.00-28.00) | 19.00 (14.00-26.00) | <0.001 | <0.001 |
| N (%) | | | | |
| Sex, N (%) |  |  | 0.128 | - |
| Female | 1009（42.20%） | 1561（44.20%） |  |  |
| Male | 1382（57.80%） | 1971（55.80%） |  |  |
| Smoking, N (%) |  |  | <0.001 | - |
| No | 1888（78.96%） | 2413（68.32%） |  |  |
| Yes | 254（10.62%） | 307（8.69%） |  |  |
| Unknown | 249（10.41%） | 812（22.99%） |  |  |
| Drinking, N (%) |  |  | <0.001 | - |
| No | 1978（82.73%） | 2529（71.60%） |  |  |
| Yes | 165（6.90%） | 191（5.41%） |  |  |
| Unknown | 248（10.37%） | 812（22.99%） |  |  |
| Hypertension, N (%) |  |  | <0.001 | - |
| No | 1235（51.65%） | 2218（62.80%） |  |  |
| Yes | 1156（48.35%） | 1314（37.20%） |  |  |
| Diabetes, N (%) |  |  | <0.001 | - |
| No | 2029（84.86%） | 3170（89.75%） |  |  |
| Yes | 362（15.14%） | 362（10.25%） |  |  |
| PAD, % |  |  | 0.017 | - |
| No | 2134(89.25%) | 3218(91.11%) |  |  |
| Yes | 257(10.75%) | 314(8.89%) |  |  |

NHHR, non-high-density lipoprotein cholesterol to high-density lipoprotein cholesterol ratio; SD, standard deviation; Apo A1, apolipoprotein A1; NEUT, neutrophil count; TC, total cholesterol; HDL-C, high-density lipoprotein cholesterol; Lp(a), lipoprotein(a); ALT, alanine aminotransferase; PAD, peripheral artery disease.

P-value*: Kruskal Wallis Rank Test for continuous variables, Fisher Exact for categorical variables with Expects<10.
