# Supplementary material for: Association between the non-high-density lipoprotein cholesterol to high-density lipoprotein cholesterol ratio and peripheral artery disease in vascular surgery inpatients aged 50 and above: a retrospective cross-sectional study
Source: Front Med (Lausanne). 2026 Jan 21;13:1739515. doi: 10.3389/fmed.2026.1739515 (PMC12868209; doi:10.3389/fmed.2026.1739515)
Supplement: Supplementary file 4 [file Table_4.docx]

Supplementary Table 4. Reasons for exclusion of patients and the number of PAD cases excluded.

| Reasons | PAD Cases/Total Cases (%) |
| --- | --- |
| Missing NHHR data | 225/2182(10.31%) |
| History of malignancy/mental disorder | 4/30(13.33%) |
| Recent myocardial infarction, stroke, or severe organ dysfunction | 1/6(16.67%) |
| Recent use of lipid-altering medications | 27/173(15.61%) |
| Total Excluded Patients | 257/2391(10.75%) |
| Total Included Patients | 314/3532(8.89%) |

PAD, peripheral artery disease; NHHR, non-high-density lipoprotein cholesterol to high-density lipoprotein cholesterol ratio.
