# Supplementary material for: Association between the non-high-density lipoprotein cholesterol to high-density lipoprotein cholesterol ratio and peripheral artery disease in vascular surgery inpatients aged 50 and above: a retrospective cross-sectional study
Source: Front Med (Lausanne). 2026 Jan 21;13:1739515. doi: 10.3389/fmed.2026.1739515 (PMC12868209; doi:10.3389/fmed.2026.1739515)
Supplement: Supplementary file 5 [file Table_5.docx]

Supplementary Table 5. Regression analysis results from multiple imputation of excluded patients.

| NHHR | Cases/n | Model 1^a^ | Model 2^b^ | Model 3^c^ |
| --- | --- | --- | --- | --- |
|  |  | OR (95%CI)  P-value | OR (95%CI)  P-value | OR (95%CI)  P-value |
| Multiple Imputation Dataset 1 | 571/5923 | 0.80 (0.69, 0.92) 0.002 | 0.88 (0.75, 1.03) 0.118 | 0.74 (0.62, 0.88) <0.001 |
| Multiple Imputation Dataset 2 | 571/5923 | 0.84 (0.73, 0.97) 0.016 | 0.94 (0.81, 1.10) 0.455 | 0.79 (0.66, 0.94) 0.007 |
| Multiple Imputation Dataset 3 | 571/5923 | 0.81 (0.70, 0.94) 0.005 | 0.88 (0.76, 1.03) 0.124 | 0.74 (0.62, 0.88) <0.001 |
| Multiple Imputation Dataset 4 | 571/5923 | 0.82 (0.71, 0.95) 0.008 | 0.92 (0.79, 1.08) 0.312 | 0.76 (0.64, 0.91) 0.003 |
| Multiple Imputation Dataset 5 | 571/5923 | 0.81 (0.70, 0.94) 0.005 | 0.91 (0.78, 1.06) 0.212 | 0.76 (0.64, 0.90) 0.002 |
| Pre-Imputation Dataset | 314/3532 | 0.83 (0.71, 0.98) 0.023 | 0.89 (0.76, 1.05) 0.170 | 0.77 (0.65, 0.93) 0.006 |

^a^No adjustment.

^b^Adjusted for age, sex, hypertension, diabetes.

^c^Adjusted for age, sex, Lp(a), Apo A1, ALT, NEUT, smoking, drinking, hypertension, diabetes.

NHHR, non-high-density lipoprotein cholesterol to high-density lipoprotein cholesterol ratio; Lp(a), lipoprotein(a); Apo A1, apolipoprotein A1; ALT, alanine aminotransferase; NEUT, neutrophil count.
