# Supplementary material for: Association between the non-high-density lipoprotein cholesterol to high-density lipoprotein cholesterol ratio and peripheral artery disease in vascular surgery inpatients aged 50 and above: a retrospective cross-sectional study
Source: Front Med (Lausanne). 2026 Jan 21;13:1739515. doi: 10.3389/fmed.2026.1739515 (PMC12868209; doi:10.3389/fmed.2026.1739515)
Supplement: Supplementary file 6 [file Table_6.docx]

Supplementary Table 6. Regression analysis results from multiple imputation of missing smoking and drinking data.

| NHHR | Cases/n | Model 1^a^ | Model 2^b^ | Model 3^c^ |
| --- | --- | --- | --- | --- |
|  |  | OR (95%CI)  P-value | OR (95%CI)  P-value | OR (95%CI)  P-value |
| Multiple Imputation Dataset 1 | 314/3532 | 0.83 (0.71, 0.98) 0.023 | 0.89 (0.76, 1.05) 0.170 | 0.78 (0.65, 0.93) 0.007 |
| Multiple Imputation Dataset 2 | 314/3532 | 0.83 (0.71, 0.98) 0.023 | 0.89 (0.76, 1.05) 0.170 | 0.78 (0.65, 0.94) 0.007 |
| Multiple Imputation Dataset 3 | 314/3532 | 0.83 (0.71, 0.98) 0.023 | 0.89 (0.76, 1.05) 0.170 | 0.78 (0.65, 0.94) 0.007 |
| Multiple Imputation Dataset 4 | 314/3532 | 0.83 (0.71, 0.98) 0.023 | 0.89 (0.76, 1.05) 0.170 | 0.78 (0.65, 0.94) 0.008 |
| Multiple Imputation Dataset 5 | 314/3532 | 0.83 (0.71, 0.98) 0.023 | 0.89 (0.76, 1.05) 0.170 | 0.78 (0.65, 0.94) 0.008 |
| Pre-Imputation Dataset | 240/2720 | 0.85 (0.71, 1.02) 0.085 | 0.91 (0.75, 1.10) 0.336 | 0.78 (0.64, 0.96) 0.021 |

^a^No adjustment.

^b^Adjusted for age, sex, hypertension, diabetes.

^c^Adjusted for age, sex, Lp(a), Apo A1, ALT, NEUT, smoking, drinking, hypertension, diabetes.

NHHR, non-high-density lipoprotein cholesterol to high-density lipoprotein cholesterol ratio; Lp(a), lipoprotein(a); Apo A1, apolipoprotein A1; ALT, alanine aminotransferase; NEUT, neutrophil count.
